# Permanent Draft Genome Sequence of *Frankia* sp. Strain ACN1^ag^, a Nitrogen-Fixing Actinobacterium Isolated from the Root Nodules of *Alnus glutinosa*

**DOI:** 10.1128/genomeA.01483-15

**Published:** 2015-12-17

**Authors:** Erik Swanson, Rediet Oshone, Stephen Simpson, Krystalynne Morris, Feseha Abebe-Akele, W. Kelley Thomas, Louis S. Tisa

**Affiliations:** University of New Hampshire, Durham, New Hampshire, USA

## Abstract

*Frankia* strain ACN1^ag^ is a member of *Frankia* lineage Ia, which are able to re-infect plants of the *Betulaceae* and *Myricaceae* families. Here, we report a 7.5-Mbp draft genome sequence with a G+C content of 72.35% and 5,687 candidate protein-encoding genes.

## GENOME ANNOUNCEMENT

Among the *Actinobacteria*, the genus *Frankia* is well known for its facultative lifestyle as a plant symbiont of dicotyledonous plants, termed actinorhizal plants, and as a free-living soil dweller (1–3). Actinorhizal plants are ecologically important pioneer community plants that are found worldwide in a broad range of ecological and environmental conditions (4). The symbiosis allows actinorhizal plants to colonize harsh environmental terrains. The genus *Frankia* has not yet been described to the species level, but it has become an area of greater interest. Four major *Frankia* lineages have been identified (5–8), and genomes for representatives from each cluster have been sequenced (9–21).

Cluster I contains two subclusters: one subcluster (Cluster Ia) represents *Frankia* strains with the ability to infect a wider range of host plants, including member of the *Betulaceae* and *Myricaceae* families, and the other subcluster (Cluster Ib) contains strains limited to *Casuarina* and *Allocasuarina* host plants. As another member of Cluster Ia, *Frankia* sp. strain ACN1^ag^ was chosen for sequencing to provide more information on this lineage and its interaction with actinorhizal plants. *Frankia* sp. strain ACN1^ag^ is a re-isolate from root nodules of *Alnus glutinosa* inoculated from an isolate of *Alnus viridis crispa* collected from Atikokan, Ontario, Canada (22, 23).

The draft genome of *Frankia* sp. strain ACN1^ag^ was generated at the Hubbard Genome Center (University of New Hampshire, Durham, NH, USA) using Illumina technology (24) techniques. A standard Illumina shotgun library was constructed and sequenced using the Illumina HiSeq2000 platform, which generated 14,474,194 reads (260-bp insert size) totaling 2,127.7 Mbp. The Illumina sequence data were assembled using CLC Genomics Workbench version 8.0.1 and AllPaths-LG version r41043 (25). The final draft assembly contained 108 contigs with an *N*_50_ of 157.4 kb. The total size of the genome is 7.5 Mbp, and the final assembly is based on 2,127.17 Mb of Illumina draft data and provided an average 220× coverage of the genome.

The high-quality draft genome of *Frankia* sp. strain ACN1^ag^ was resolved to 108 contigs consisting of 7,505,639 bp with a G+C content of 72.35%. The assembled *Frankia* sp. strain ACN1^ag^ genome was annotated via the Integrated Microbial Genomes (IMG) platform developed by the Joint Genome Institute, Walnut Creek, CA, USA (26, 27), and resulted in 5,687 candidate protein-encoding genes, 45 tRNA genes, and 2 rRNA regions.

### Nucleotide sequence accession numbers.

This whole-genome shotgun project has been deposited at DDBJ/EMBL/GenBank under the accession number LJPA00000000. The version described in this paper is the first version, LJPA01000000.
